# High Potential of Bacterial Adhesion on Block Bone Graft Materials

**DOI:** 10.3390/ma13092102

**Published:** 2020-05-01

**Authors:** Themistoklis Nisyrios, Lamprini Karygianni, Tobias Fretwurst, Katja Nelson, Elmar Hellwig, Rainer Schmelzeisen, Ali Al-Ahmad

**Affiliations:** 1 Department of Oral and Craniomaxillofacial Surgery, Center for Dental Medicine, University Medical Center Freiburg, Faculty of Medicine, 79106 Freiburg, Germany; tobias.fretwurst@uniklinik-freiburg.de (T.F.); katja.nelson@uniklinik-freiburg.de (K.N.); rainer.schmelzeisen@uniklinik-freiburg.de (R.S.); 2 Clinic for Preventive Dentistry, Periodontology and Cariology, Center for Dental Medicine, University of Zurich, 8032 Zurich, Switzerland; lamprini.karygianni@zzm.uzh.ch; 3 Department of Operative Dentistry and Periodontology, Center for Dental Medicine, Medical Center, Faculty of Medicine, University of Freiburg, 79106 Freiburg, Germany; elmar.hellwig@uniklinik-freiburg.de (E.H.); ali.al-ahmad@uniklinik-freiburg.de (A.A.-A.)

**Keywords:** initial bacterial adhesion, block bone grafts, chlorhexidine (CHX), scanning electron microscopy (SEM), bone graft bacterial contamination

## Abstract

Bone graft infections represent a challenge in daily clinics, resulting in increased patient discomfort and graft removal. The aim of this study was to investigate the initial adhesion of five representative pathogens on three different block bone graft materials (xenogeneic, alloplastic and allogeneic) and to assess if chlorhexidine (CHX) can effectively control the initial bacterial adhesion. Three different block bone grafting materials (Tutobone®, Endobon® and human spongiosa) were incubated with *Escherichia coli*, *Staphylococcus aureus*, *Streptococcus mutans*, *Enterococcus faecalis* and *Pseudomonas aeruginosa* in the presence or absence of 0.2% CHX solution. Bacterial adhesion was assessed by the direct counting of the colony-forming units (CFUs) and visualized by scanning electron microscopy (SEM). Overall, the selected bacterial species adhered successfully to all tested bone replacement scaffolds, which showed similar bacterial counts. The lg CFU values ranged from 5.29 ± 0.14 to 5.48 ± 0.72 for *E. coli*, from 4.37 ± 0.62 to 5.02 ± 0.48 for *S. aureus*, from 4.92 ± 0.34 to 4.95 ± 0.21 for *S. mutans*, from 4.97 ± 0.40 to 5.22 ± 0.13 for *E. faecalis* and from 4.23 ± 0.54 to 4.58 ± 0.26 for *P. aeruginosa.* CHX did not interfere with initial microbial adhesion, and yet it killed all adhered bacterial cells. Thus, CHX can be used to prevent subsequent biofilm infections.

## 1. Introduction

Teeth extractions, periodontal disease, cyst resection, trauma, and benign or malignant tumors of the facial skeleton result inevitably in bone deficiencies and in unfavorable conditions for the rehabilitation of the partially or totally edentulous patient. Different grafting procedures and modifications have been proposed to rehabilitate small or larger bone defects [1,2]. For severe bone deficiencies, autologous bone blocks facilitate the adequate increase in bone volume because they possess the essential properties for new bone formation, namely osteogenicity, osteoconduction, and osteoinduction. On the downside, the morbidity of the donor site and the limited availability remains a major disadvantage for the clinician and for the patient, restricting the use of autologous bone blocks. Due to these limitations, oral and maxillofacial as well as orthopedic surgeons have opted for the alternative use of allogeneic, xenogeneic or alloplastic block bone graft materials [3,4].

Allogeneic grafts represent the second most common bone transplantation worldwide, they are mostly cadaveric in origin and originate rarely from living donors, due to the higher risk of disease transmission, antigenicity and immune reaction [5,6]. They are usually either demineralized freeze-dried (DFDBA) or mineralized freeze-dried (FDBA) preparations. The demineralization process increase their osteoinductive capabilities. However, the irradiation and the rigorous process to remove potential antigens and pathogens leads to a low concentration of proteins, so that a minimal osteoinductive capability is assumed. Therefore, allografts act primarily as scaffolds with osteoconductive properties [7,8].

Xenogeneic graft is a bone tissue from nonhuman species. It is mainly of bovine origin, followed by grafts from porcine or equine sources. They usually undergo a demineralization and further processing. Different processing methods of xenografts result in different clinical material specifications, among which the resorption rate of the graft serves as the most important parameter [9]. The remaining minerals act as a scaffold for the formation of the new bone that possesses only osteoconductive properties. Their combination with growth factors or the impregnation with bone marrow aspirate (BMA) can stimulate bone osteogenesis [10]. Charwat-Pessler et al. combined micro-computed tomography (μ-CT) with Raman spectroscopy in order to present a non-invasive method to assess the ability of bone graft materials to promote new bone formation [11].

Alloplastic bone substitutes are artificial bone grafting materials and include hydroxyapatite, calcium phosphates, calcium sulfate, collagen, and polymers [12,13]. They can be treated to be resorbable or non-resorbable, to have various pore sizes and various forms. They have only osteoconductive properties but they can also be combined with bone morphogenetic proteins-BMP’s or be enriched with BMA and thus become osteoinductive and/or osteogenic, serving as an ambitious alternative to autologous graft [4]. On the upside, the absence of risk for disease transmission, the lack of antigenicity and their supply in unlimited quantities favors the use of alloplastic materials [4,14].

The major and most important complications following a block bone grafting are the soft tissue dehiscence and the consequential exposure and infection of the bone graft, resulting in a partial or a total loss of the grafted material. The bone graft acts temporarily or permanently as a foreign body, initially free of vascularization and characterized by a reduced resistance against bacteria [15]. Busscher et al. considered sterile surgery to be a myth [16]. Indeed, according to the most researchers, the contamination of the graft cannot be prevented and occurs either during surgery or in the wound healing phase through the wound incision or via the blood [17,18]. Furthermore, various studies estimate the incidence of bacterial infection of the transplanted bone graft material between 0.7% and 13%, implying that the bacterial contamination of bone graft does not necessarily lead to an infection [19,20]. 

The bacteria causing these infections are mostly organized in well-structured, surface-associated biofilms. Several bacterial pathogens have been associated with bone grafting procedures in oral, maxillofacial and orthopedic surgery. The biofilm revealed on bone surface contains predominantly Gram-positive cocci, namely staphylococci. The Gram-negative rods also play an important role in the biofilm formation [21]. As it is well known that biofilm formation begins with the initial microbial adhesion of the biomaterial surface, preventing this initial step would protect diverse surfaces from biomaterial-associated biofilm infections [22,23].

The use of chlorhexidine (CHX) as mouth rinse preoperatively, as well as the bleaching of bone debris collected intraoperatively with CHX, has been shown to reduce the microbiological burden and the risk of infection, thereby increasing the viability rates of the implanted graft materials and the success rates of placed dental implants [24]. Pommer et al., could demonstrate that the use of CHX solution resulted in the highest percentage reduction (99.97% mean value) of bacterial colony-forming units (CFUs) in bone grafts when compared with other disinfecting and antibiotic agents like Povidone-Iodine, Rifamycin, Clindamycin, liquid soap, Tetracycline, Ethanol and Bacitracin. For safe use in bone grafting surgery, a decontamination protocol with CHX 1% inducing no cytotoxic effects on osteoblast growth and differentiation was proposed for 15–30 s. [25].

The aim of this study was (i) to investigate the initial adhesion of five bacterial species on three different block bone graft materials (xenogeneic, alloplastic and allogeneic) that are widely used to reconstruct bone defects and (ii) to assess if CHX solution can prevent the initial bacterial colonization. 

## 2. Materials and Methods

### 2.1. Selection and Preparation of Biomaterials

**Figure 1:**
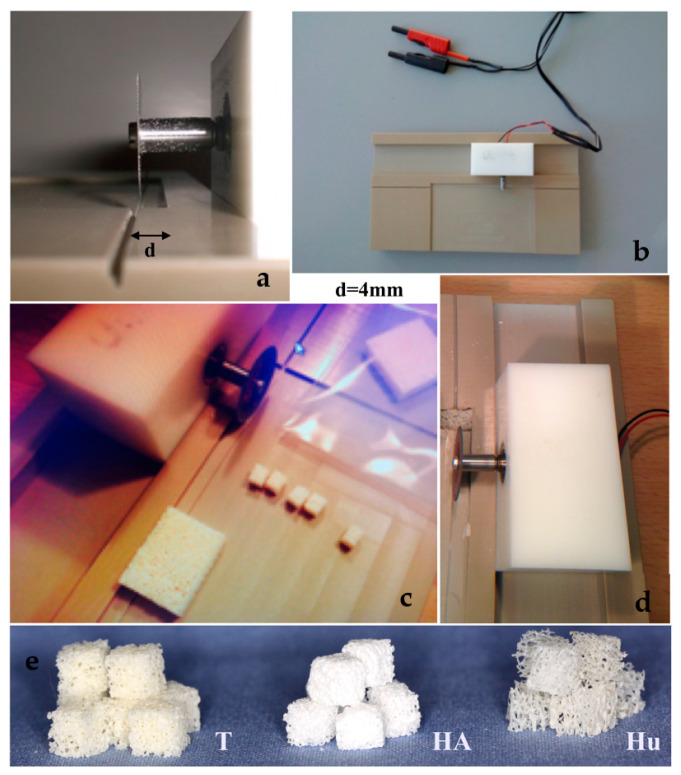
(**a**–**d**) Sawing the block bone grafts to 4mm edge cubes using a saw machine, designed specifically for the purposes of the study, (**e**) 4 mm bone cubes of three different bone graft materials (T = Tutobone_®_-Xenogeneic graft, HA = Hydroxylapatite alloplastic graft-Endobon^®^, Hu = Human Spongiosa).

Three different block bone graft materials were purchased. A graft of bovine origin, namely Tutobone® (Tutogen, Neuenkirchen am Brand, Germany) was used as xenogeneic graft, human spongiosa (German Institute for cell and tissue replacement, Berlin, Germany) was used as allogeneic graft, while an alloplastic hydroxyapatite bone block, namely Endobon® (Zimmer Biomet, Warsaw, IN, USA) was tested as synthetic bone graft. All three materials are widely used in bone reconstructive operations in oral and maxillofacial as well as orthopedic surgery. Prior to the assays, the block bone graft materials were sawed in cubes with a 4-mm edge each, using a saw machine that was designed in the Department of Operative Dentistry and Periodontology, University of Freiburg, Freiburg, Germany, for the purposes of this study (Figure 1). For disinfection, the samples were immersed in 70% alcohol solution for 2 d, and then they were rinsed meticulously with 0.9% saline solution (NaCl) and stored in sterile Cellstar®-cell tissue culture plates (Greiner Bio-One International GmbH, Kremsmünster, Austria). 

### 2.2. Bacterial Strains

The bone graft cubes were incubated with the bacterial strains *E. coli* ATCC 29522, *S. aureus* ATCC 25923, *S. mutans* DSM 20523, *E. faecalis* T9 and *P. aeruginosa* ATCC 27853. The strains *E. coli*, *S. aureus* and *P. aeruginosa* were provided from the nonprofit organization American Type Culture Collection (ATCC, Manassas, VA, USA), *S. mutans* was supplied from the German Collection of Microorganisms and Cell Cultures (Leibniz Institute-DSMZ, Braunschweig, Germany), while the strain *E. faecalis* T9 described by Maekawa et al. [26] was obtained by the Department of Medical Infectiology, University Freiburg, Freiburg, Germany. All selected strains are considered as representative pathogens detected in bone graft infections [27,28].

### 2.3. Quantification of the Initial Microbial Adhesion

**Figure 2:**
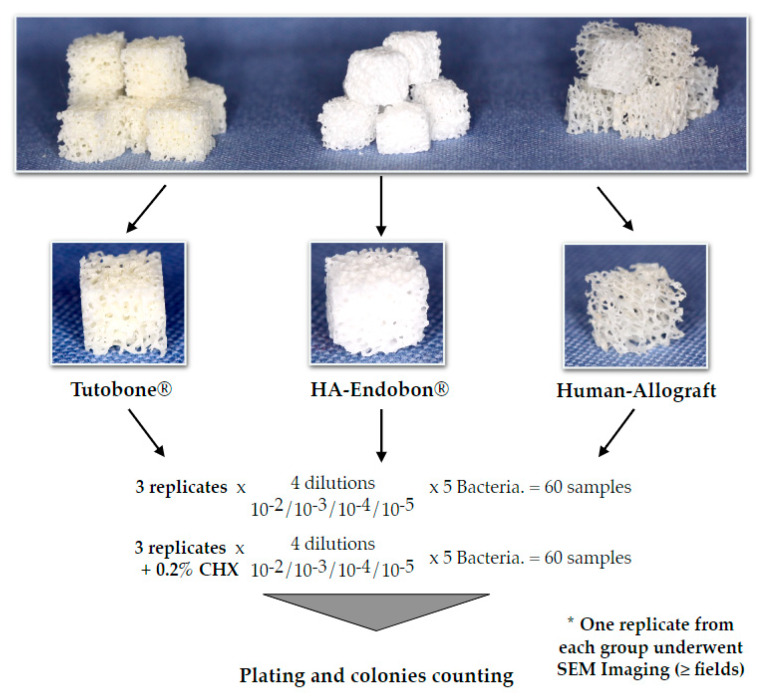
Depiction of the experimental flow diagram. The experiments for each bacterium and bone graft group were repeated three independent times. In total, 360 cube-shaped samples of the three different grafting materials were used to evaluate the bacterial growth on bone, in the presence or absence of chlorhexidine (CHX) solution in a quantitative pattern.

The experimental design was performed three times for each microorganism and material. (Figure 2). Briefly, pure cultures of *E. coli*, *S. aureus*, *S. mutans*, *E. faecalis* and *P. aeruginosa* were incubated in 5 mL of tryptic soy broth (TSB) overnight at 37 °C. On the next day, the bacterial cultures were centrifuged at 4000 g for 10 min. A total of 4 mL of the medium were aspirated, so that 1 mL of the medium with the bacterial sediment could remain in the tube. The remaining medium was diluted with 4 ml 0.9% NaCl, and 1 ml of this solution was added to a tube with 9 mL 0.9% NaCl. Subsequently, serial dilutions thereof (10^−2^, 10^−3^, 10^−4^ and 10^−5^) were made for each microorganism. In order to test the affinity and the adherence potential of the bacterial cells on the bone grafts, four cubes of disinfected bone graft material were incubated with bacteria for 2 h in 37 °C, then transferred to small tubes using sterile forceps to avoid a cross-infection and sonicated in 0.9% NaCl for 2 min at a 70% digital output to allow for the release of the adherent bacteria, which were then plated on Columbia blood agar plates (Oxoid Ltd, Basingstoke, UK). The plates were left in the incubator at 37 °C with 5% CO_2_, and, after 2 d, the colonies were counted using a digital colony counter device BZW 40 (Xylem Analytics Germany Sales GmbH & Co. KG, Weilheim in Oberbayern, Germany). In order to test the efficacy of CHX solution, we performed the same procedure using CHX-treated bone grafts. Two percent CHX solution was supplied from the central pharmacy of the University Hospital of Freiburg (Apotheke des Universitätsklinikums Freiburg, Freiburg, Germany) and was then diluted with sterile water to a 0.2% CHX solution. Thus, after the incubation of the bone cubes with 5 different bacterial solutions for 2 h, the contaminated bone grafts were incubated in 0.2% CHX solution for 2 min, followed again by the incubation, sonication, plating and counting of the bacterial colonies after incubation of the plates for 2 d as described above. The described procedure was conducted independently three times for each microorganism and material group, so that a total of 360 bone cubes could be tested for all bone graft materials and all bacteria. 

### 2.4. Scanning Electron Microscopy—SEM

For scanning electron microscopy (SEM), the same procedure of incubating the three different bone graft materials with five microorganisms (*E. coli*, *S. aureus*, *S. mutans*, *E. faecalis* and *P. aeruginosa*) was performed. The untreated groups were incubated for 2 h at 37 °C to allow for bacterial adhesion on the bone surface. In the CHX-treated group, the inoculated bone graft cubes were soaked in 0.2% CHX solution for 2 min and incubated for 2 h at 37 °C with 5% CO_2_. For SEM, the bone scaffolds were fixed in 8% formaldehyde for 3 d at 4 °C and dehydrated in graded alcohol (30%, 50%, 70%, 80%, 90%, one time each and two times in 99.8% for 1 h). According to standard procedure, after critical point drying (Critical Point Dryer CPD 030, Bal-Tec, Walluf, Germany) using liquid carbon dioxide, the samples were sputtered with gold in a SCD 050 coater (Bal- Tec, Walluf, Germany). The samples were examined with the aid of a Zeiss Leo 435 VP scanning electron microscope (Leo Electron Microscopy Ltd Cooperation Zeiss Leica, Cambridge, UK) at 10 kV and representative images were captured in various magnifications. 

### 2.5. Statistical Analysis

The statistical analysis was performed with STATA 15.1 (StataCorp LLC, College Station, TX, USA). A one-way analysis of variance and adjustment of *p*-values with Bonferroni’s multiple comparison correction test was applied. A *p*-value of < 0.05 (two-sided) was considered statistically significant.

## 3. Results

### 3.1. All Bone Graft Materials Presented a Comparable Initial Bacterial Adhesion for the Five Selected Bacterial Species

**Figure 3:**
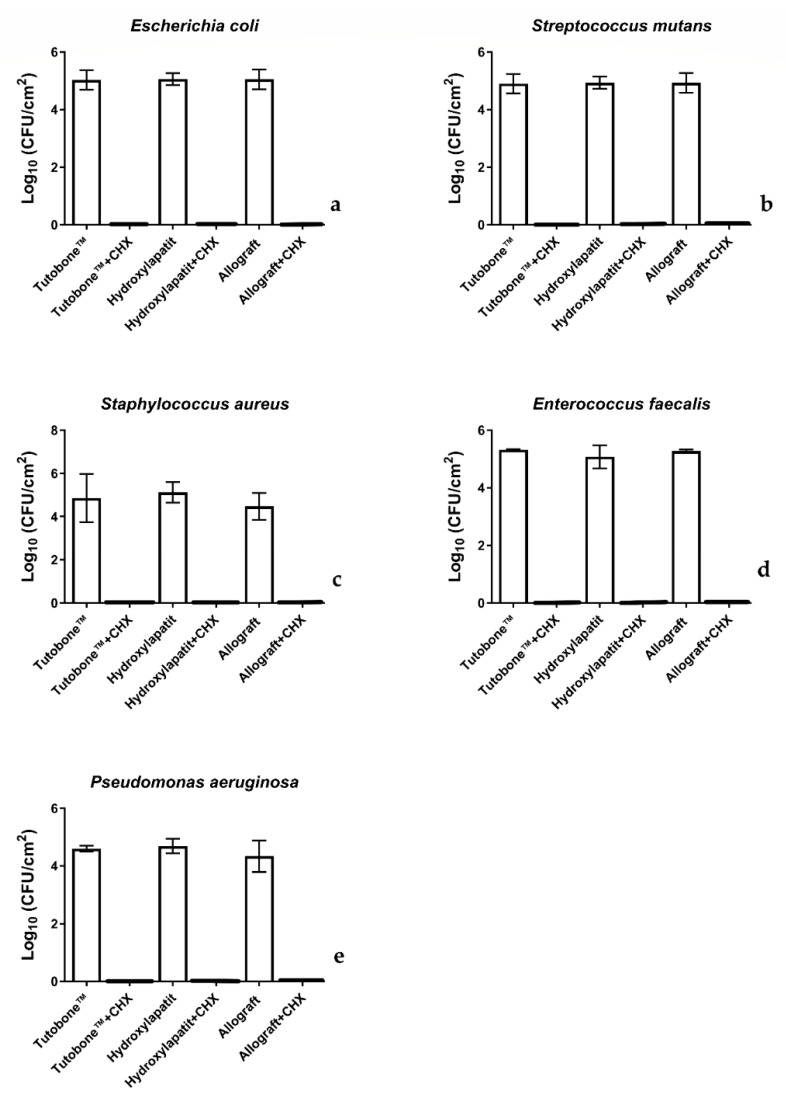
Boxplots demonstrating the colony-forming units (CFUs) of *E. coli*, *S. aureus*, *S. mutans, E. faecalis* and *P. aeruginosa* on the tested bone graft materials with or without the application of 0.2% CHX solution. As shown in diagram, no bacterial colonies were detected after treatment with 0.2% CHX solution. (**a**) *E. coli*, (**b**) *S. mutans*, (**c**) *S. aureus*, (**d**) *E. faecalis* and (**e**) *P. aeruginosa*.

In total, five bacterial strains were tested for the adhesion on three different bone graft materials, with or without treatment with 0.2% CHX solution. Figure 3 shows the number of CFUs determined for each microorganism attaching to each bone scaffold after a 2-h incubation. The CFUs are presented on a lg scale per square centimeter (lg/cm^2^). In regard with the non-CHX-treated samples, the initial bacterial adhesion of all five tested microorganisms was similar between the three bone graft materials (xenogeneic, allogeneic, synthetic). In particular, the untreated control for the xenogeneic bone graft material Tutobone® revealed a lg CFU value of 5.48 ± 0.72 for *E. coli*, 4.74 ± 1.12 for *S. aureus*, 4.92 ± 0.34 for *S. mutans*, 5.22 ± 0.13 for *E. faecalis* and 4.49 ± 0.11 for *P. aeruginosa*, respectively. The corresponding values for the alloplastic bone graft material Endobon® were 5.31 ± 0.12 for *E. coli*, 5.02 ± 0.48 for *S. aureus*, 4.95 ± 0.21 for *S. mutans*, 4.97 ± 0.40 for *E. faecalis*, 4.58 ± 0.26 for *P. aeruginosa*, whereas the allogeneic graft materials yielded lg CFU values of 5.29 ± 0.14 for *E. coli*, 4.37 ± 0.62 for *S. aureus*, 4.94 ± 0.34 for *S. mutans*, 5.17 ± 0.56 for *E. faecalis* and 4.23 ± 0.54 for *P. aeruginosa*, respectively. 

### 3.2. Pretreatment of the Bone Graft Material with CHX Killed the Adhered Bacteria

Figure 3 displays the CFUs counts of the initial bacterial adhesion following a 2-h incubation of the tested bone grafts with five different microorganisms and treatment with 0.2% CHX solution for 2 min. Contrary to the untreated group, no bacterial colonies were detected after treatment with 0.2% CHX solution, revealing a high-level of antimicrobial activity of this disinfectant. 

### 3.3. The Morphology of the Scaffolds Allowed for the Organization of the Adherent Bacterial Cells in Grape-Like Clusters

**Figure 4:**
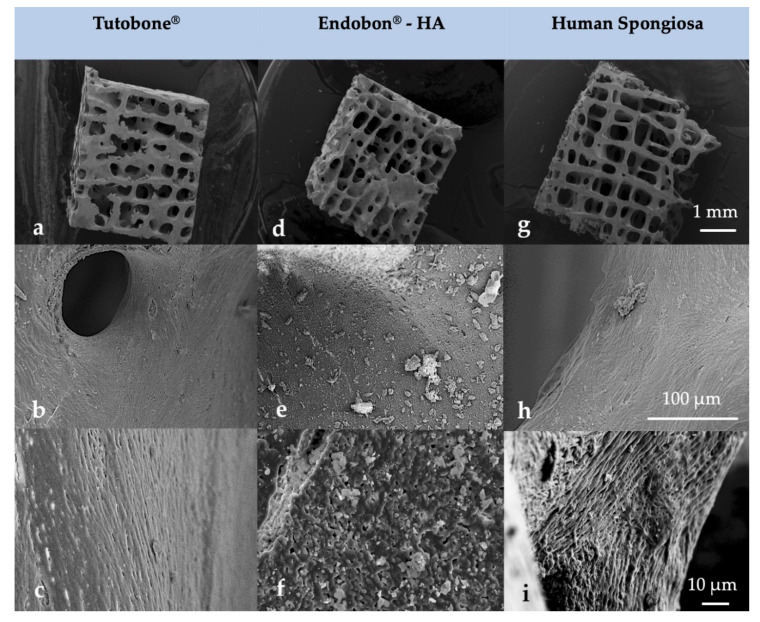
Surface morphology of the tested block bone graft materials (**a**–**c**) Tutobone^®^, (**d**–**f**) Endobon^®^-HA and (**g**–**i**) Human spongiosa in a magnification of ×25 (top row—(**a**,**d**,**g**)), ×1000 (middle row—(**b**,**e**,**h**)) and ×3000 (bottom row—(**c**,**f**,**i**)) using scanning electron microscopy (SEM) analysis. The bar represents 1 mm, 100 μm and 10 μm, respectively.

**Figure 5:**
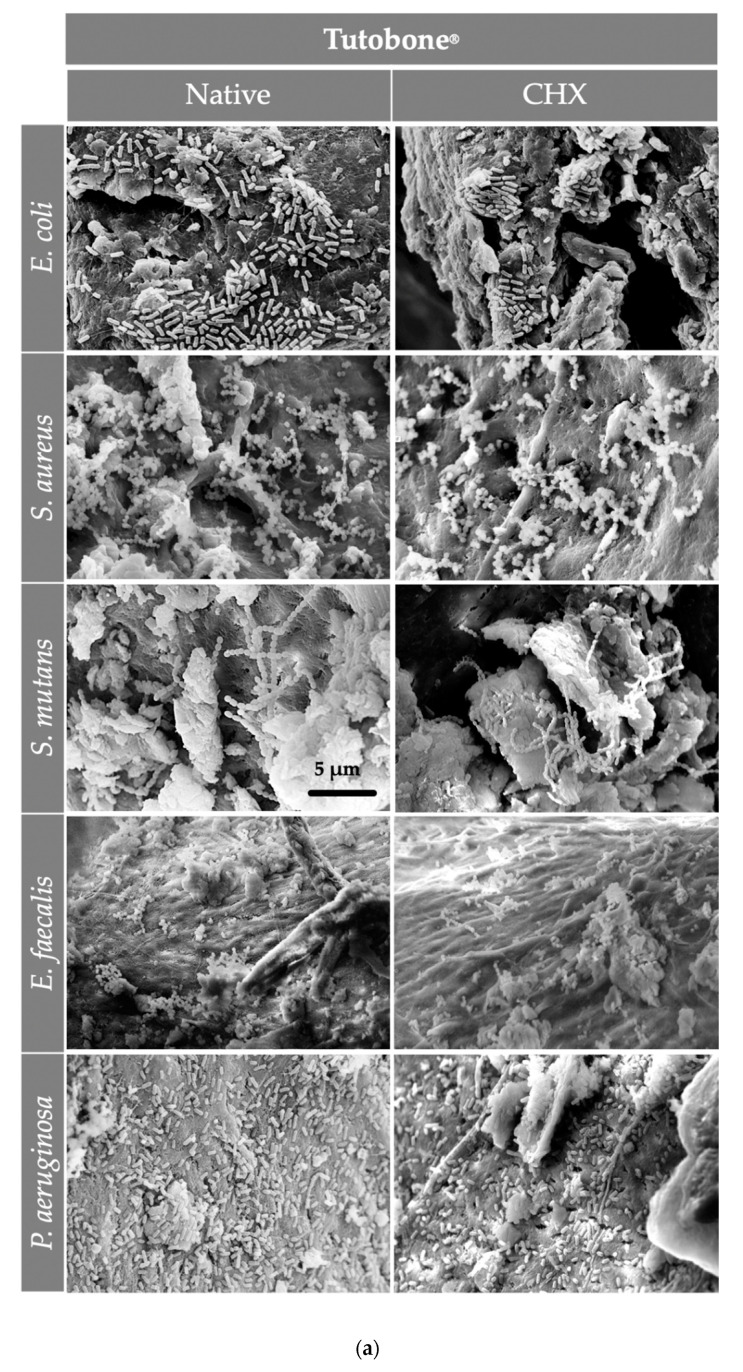

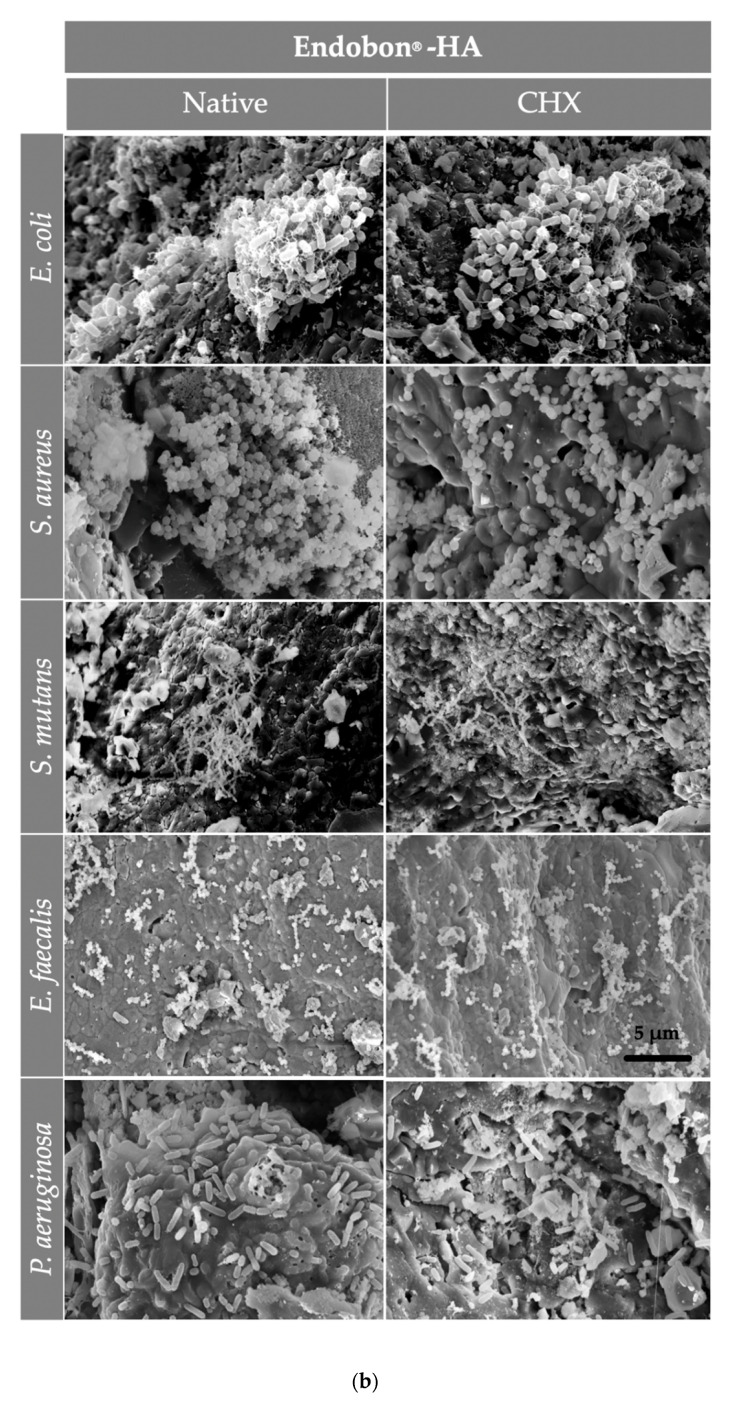

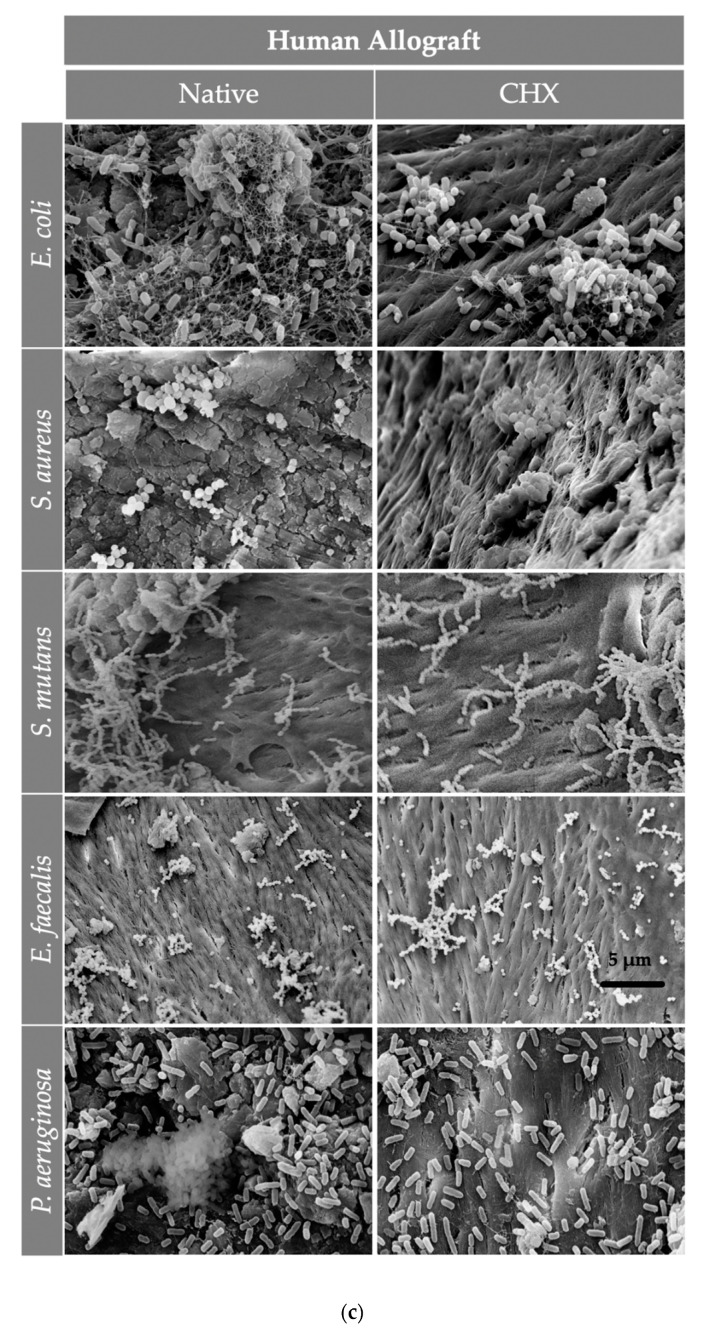
The SEM assessment for all bone graft materials [(**a**) Tutobone_®_, (**b**) HA-Endobon_®_ and (**c**) Human Spongiosa] and bacteria strains in presence or absence of CHX solution 0.2% in a magnification of ×5000 is depicted. The micrograph shows a successful adhesion of the microorganisms on the bone scaffold with formation of bacterial clusters or chains on the planar surfaces as well as in the topographic niches. The bar represents 5 μm.

After the visualization of the initial bacterial adhesion on three different bone grafts (xenogeneic, allogeneic, synthetic) using SEM, we observed that all tested pathogens (*E. coli*, *S. aureus*, *S. mutans*, *E. faecalis*, *P. aeruginosa*) adhered successfully on the tested surfaces of all three bone graft materials, independently of the treatment with CHX. Moreover, there was no significant difference in the bacterial adherence between the three bone graft materials. At higher magnifications, it was evident that the bacterial cells of *E. coli*, *S. aureus*, *E. faecalis* and *P. aeruginosa* frequently formed grape-like clusters, while the bacterial cells of *S. mutans* grew in long chains. Both planar surfaces and niches of the bone grafts were colonized by all tested pathogens. Interestingly, the bacterial cells of *E. coli* produced flocs, which were embedded in a net formed by the polymeric matrix (Figure 4 and Figure 5). After examining the SEM images, we could conclude that the adherent bacteria were killed by the CHX solution and therefore no CFUs could be visualized on the bone graft surfaces after treatment with 0.2% CHX. 

## 4. Discussion

Sterile bone graft transplantation in the mouth cavity is a utopia. Contamination can occur either during the harvesting process and placement into the recipient site or during the healing phase, hematogenously or due to wound dehiscence [16]. Allogeneic, xenogeneic and alloplastic bone graft materials are all harvested or manufactured and sterilized extracorporeal. This means that they pose a risk of contamination during the graft placement or healing process [16]. The augmentation procedure may be associated with various soft and hard tissue complications [15,29,30]. The effective modification of the widely used bone graft materials to resist bacterial infections is a challenge in the research field of biomaterials in oral and maxillofacial surgery. A possible way to achieve this could be the prevention of the biofilm development on bone graft surfaces. The current biofilm research focuses, among other things, on the emergent properties of microbial communities and their resistance to diverse antimicrobial agents [31]. The aim of the present study was to evaluate the in vitro adherence of five potentially pathogenic bacterial strains on three different bone graft materials (allogeneic, xenogeneic and alloplastic) with or without CHX treatment.

The autogenous block bone grafts are considered as the gold standard for bone reconstruction, especially for severe maxillomandibular atrophy [2,32]. For the mandible, techniques such as the reconstruction with block bone grafts and the placement of dental implants appears to be preferable than the transposition of the inferior alveolar nerve. This is due to the fact that the latter technique is characterized by a high incidence of nerve damage and the risk of mandible fracture [33]. The survival rates of dental implants placed on augmented areas are considered to be similar to those related with non-grafted areas [34,35,36]. However, the morbidity of the donor site and the limited availability of autografts remain their major drawbacks. Moreover, excessive graft resorption, up to 50% of the graft volume, regardless of the donor site or harvesting technique, can be another concern [37]. In an earlier report, Nyström et al. observed high rates of width reduction after the use of iliac crest onlay bone grafts from 12.2 to 8.7 mm at 12 months [38]. The bone resorption is considered to be a part of the natural bone remodeling during the healing phase [39]. Nevertheless, bone grafts are more likely to maintain the volume of the initial augmentation when compared to guided bone regeneration (GBR) techniques [40]. Due to these limitations concerning excessive augmentation techniques, clinicians opt for the use of allogeneic, xenogeneic or alloplastic bone graft materials. 

According to several studies, the use of allogeneic block bone grafts represents an efficient, with predictable results alternative to autogenous grafts in order to reconstruct severely atrophic jaws. The groups of Barone et al. and Nissan et al. described a success rate close to 100% for allografts (99.20% and 100%, respectively) [41,42]. Several studies have reported the use of xenogeneic bone grafts for the augmentation of the atrophied jaw ridge. Researchers described a prolonged healing period with new bone formation occurring after 6–10 months. Moreover, the crucial role of the periosteum has been highlighted. Thus, its preservation with minimal invasive augmentation techniques, such as the flapless or tunneling technique, or the absence of periosteum release incisions promotes osteogenesis around or inside the grafted material [34,43]. The use of coralline porous hydroxyapatite block (PBHA) as a bone graft substitute in orthognathic and craniomaxillofacial surgery first described by Woldford et al. [44]. In their report, Kattimani et al. published promising results in the reconstruction of large mandibular defects with PBHA afterthe resection of benign tumors [45]. Additionally, several histomorphometrical studies showed that bone growth through PBHA grafts was completed with the maturation of the ingrown bone in 4 months [46,47]. However, the augmentation may be associated with various soft and hard tissue complications. According to Chaushu et al., the most common complications following a block bone graft transplantation are wound dehiscence and infection of the graft recipient site. This can subsequently lead to a partial or total loss of the transplant with serious complications, such as neurosensory disturbances and resorption of the grafted material [15,29,30]. 

Chlorhexidine (CHX) is a disinfectant with broad spectrum against pathogen microorganisms and has been widely used worldwide in medicine and dentistry since 1954. It can be used preoperatively as well as intra- or postoperatively [48]. The use of CHX as a mouth rinse preoperatively [49], and the CHX-induced bleaching of bone debris collected intraoperatively, has been shown to effectively reduce the microbiological burden and thus the risk of infection. As a result, there is an increase in the viability rates of the implanted grafts and the success rates of dental implants [24,50,51]. Currently, there is a need to establish disinfection protocols with high antimicrobial effectiveness that have no impact on the osteogenic capacity of the bone graft. According to a systematic review of Pommer et al., the CHX treatment of bone grafts resulted in the highest bacterial reduction (99.97%) when compared to other disinfectants and antibiotics. A decontamination protocol with 1% CHX for 15–30 s had no cytotoxic effects on osteoblast growth and can be safely used in bone transplantation surgery [25]. Moreover, Verdugo et al. described effective disinfecting results with no alterations in the osteoblast phenotype after the exposure of the bone transplant to 0.2% CHX and 1% CHX for 1 min and 30 s, respectively [52]. The usual side effects of CHX include taste alteration, skin irritation, teeth discoloration and allergic reactions, which could range from a harmless hypersensitivity to a life-threatening anaphylaxis [48]. Furthermore, CHX seems also to have an ototoxic effect when utilized in ear operations but is supposed to be safe during the pregnancy [53]. According to a recent study from Cieplik et al., the liberal and uncontrolled long-term use of CHX as disinfectant could induce the establishment of resistant bacterial species [54]. 

Staphylococci, especially *S. aureus*, is regarded as the most pathogenic bacterial species of the bone tissue [21]. *E. faecalis* and *S. mutans* were isolated among others from bone particles collected intraorally during implant-site preparation [55]. The pathogens *P. aeruginosa*, *S. aureus*, *E. coli* and *E. faecalis* were reported to be increasingly resistant to a wide range of antibiotics [56,57] and have been frequently isolated from diverse biomaterial-associated infections [16,58,59,60]. Regarding the bone graft material, diverse factors such as surface roughness, chemistry and hydrophobicity influence the bacterial adherence. Consequently, modifications of the bone graft surface may affect the bacterial adhesion and thus, the related risk of infection. Generally, porous materials have a significant higher rate of infection in comparison with dense materials [17]. The presence of highly resistant bacteria in the complex bone graft structure and the absence of vascularization in the block bone can explain the poor effectiveness of antibiotic treatment. The similar patterns in the bacterial colonization of infected bone grafts in chronic suppurative osteomyelitis were revealed in an earlier study [21]. If a treatment is required, surgical removal of the implanted material or a thorough debridement of the infected bone seems to be the most efficient means to eradicate the source of infection [21,61].

In the present study, the block bone grafts of the untreated groups allowed for the initial adhesion of all five tested pathogens, revealing a comparable affinity to Gram-positive and Gram-negative bacteria. In two earlier studies, the initial bacterial adhesion on 3D-scaffolds used as bone replacement materials was tested using the same microbial methods as those applied in the present study [62]. The results revealed a high risk of biomaterial infection due to increased bacterial adhesion to the tested biomaterials. As shown by SEM, the tested pathogens attached to the bone scaffolds in the CHX-treated groups, but all adherent bacteria were eventually killed. These results indicate the ability of CHX to reduce the infection risk of bone graft materials, even in low concentrations up to 1% [25]. Interestingly, the results of the CHX-treated group confirmed the high affinity of bacteria to all graft materials. More than 60% of all hospital-acquired microbial infections are caused by biofilms [16,63,64]. Once a biofilm is formed on infected implant materials, a treatment is hardly possible due to the high resistance of microorganisms against antimicrobials, as compared with their planktonic counterparts [65,66]. The mechanisms of resistance in biofilms include different mechanisms, such as the stress response of bacterial cells, the restricted penetration of antimicrobials, and the presence of microenvironments within the biofilm [67]. The prevention of initial adhesion actually means the prevention of biofilm formation and saves implant patients from threatening infections as well as high additional costs for subsequent treatment [16].

A limitation of the present study is that we assessed colonization by examining only one microorganism each time. In real conditions, more than one pathogen is involved in an infection [28]. For the quantification of the bacterial counts and visualization, CFUs and SEM were applied, respectively. Only viable cultivable bacteria are counted using CFUs, while viable but nonculturable bacteria cannot be counted. Another drawback of the CFU method is that clumps of bacterial cells can be miscounted as single colonies. Moreover, the CFUs are counted 1–3 d after platting, making this technique unsuitable for serial longitudinal studies [68]. SEM is a visualization tool and allows for the optical assessment of bacterial adherence on the bone graft surface. Using SEM has the disadvantage that only limited areas of the bone surface can be examined [28]. The three-dimensional morphological features of the bone graft material, with planar surfaces and deep niches, posed challenges for its visual assessment. The aforementioned issues, including a possible synergistic effect of ultrasonication with CHX, could be overcome in future studies using additional viability tests like live/dead staining. 

The management of a postoperative complication after bone transplantation, such as wound dehiscence and infection of the implanted material, is of high importance in the daily clinical practice. Innovative techniques involving antibiotic incorporation and delivery via carriers of different composition are already being used. However, as antibiotic resistance becomes more common, advanced novel strategies that do not rely on obsolete antibiotic mechanisms are being developed. Current and future research involves dispersal agents, bacteriophage-releasing materials, antibiofilm surface modifications and coatings and bacterial interference [69]. In a recent in vivo study, Carinci et al. showed alterations of the microbiota causing peri-implant disease and a significant reduction in the total bacterial loading at the level of peri-implant tissue after coating of the internal chamber of a dental implant with an alcoholic solution containing 1% CHX and polysiloxane oligomers [70]. Various other disinfectants, including CHX, triclosan, and benzalkonium chloride have bactericidal effects and are used widely to coat biomedical devices, in order to prevent the adhesion and growth of pathogens [12,21,71]. Treatment of the bone grafts for 2 min with 0.2% CHX resulted in successful killing of bacteria that initially adhered to the bone scaffolds and should be taken into consideration when establishing new disinfection protocols.

## 5. Conclusions

In conclusion, all five bacterial pathogens showed a high adhesion potential on the three tested block bone grafts surfaces. The disinfectant CHX showed a high antimicrobial activity when used in a concentration of 0.2% to treat block bone graft surfaces for 2 min. Further in situ studies on the disinfection of block bone graft materials are needed to reveal additional clinically relevant findings, which will lead to the establishment of effective disinfection protocols.
